# Identification of B-cell epitopes in an antigen for inducing specific class of antibodies

**DOI:** 10.1186/1745-6150-8-27

**Published:** 2013-10-30

**Authors:** Sudheer Gupta, Hifzur Rahman Ansari, Ankur Gautam, Open Source Drug Discovery Consortium, Gajendra PS Raghava

**Affiliations:** 1 Bioinformatics Centre, CSIR-Institute of Microbial Technology, Chandigarh 160036, India; 2 Open Source Drug Discovery Unit, Council of Scientific and Industrial Research (CSIR) Anusandhan Bhawan, 2 Rafi Marg, New Delhi 110001, India

**Keywords:** Support vector machine, Prediction, Antibody, Class-specific, B-cell epitope, Isotype

## Abstract

**Background:**

In the past, numerous methods have been developed for predicting antigenic regions or B-cell epitopes that can induce B-cell response. To the best of authors’ knowledge, no method has been developed for predicting B-cell epitopes that can induce a specific class of antibody (*e.g.,* IgA, IgG) except allergenic epitopes (IgE). In this study, an attempt has been made to understand the relation between primary sequence of epitopes and the class of antibodies generated.

**Results:**

The dataset used in this study has been derived from Immune Epitope Database and consists of 14725 B-cell epitopes that include 11981 IgG, 2341 IgE, 403 IgA specific epitopes and 22835 non-B-cell epitopes. In order to understand the preference of residues or motifs in these epitopes, we computed and compared amino acid and dipeptide composition of IgG, IgE, IgA inducing epitopes and non-B-cell epitopes. Differences in composition profiles of different classes of epitopes were observed, and few residues were found to be preferred. Based on these observations, we developed models for predicting antibody class-specific B-cell epitopes using various features like amino acid composition, dipeptide composition, and binary profiles. Among these, dipeptide composition-based support vector machine model achieved maximum Matthews correlation coefficient of 0.44, 0.70 and 0.45 for IgG, IgE and IgA specific epitopes respectively. All models were developed on experimentally validated non-redundant dataset and evaluated using five-fold cross validation. In addition, the performance of dipeptide-based model was also evaluated on independent dataset.

**Conclusion:**

Present study utilizes the amino acid sequence information for predicting the tendencies of antigens to induce different classes of antibodies. For the first time, *in silico* models have been developed for predicting B-cell epitopes, which can induce specific class of antibodies. A web service called IgPred has been developed to serve the scientific community. This server will be useful for researchers working in the field of subunit/epitope/peptide-based vaccines and immunotherapy (http://crdd.osdd.net/raghava/igpred/).

**Reviewers:**

This article was reviewed by Dr. M Michael Gromiha, Dr Christopher Langmead (nominated by Dr Robert Murphy) and Dr Lina Ma (nominated by Dr Zhang Zhang).

## Background

Innate and adaptive immune responses are the two main arms of host immune system to combat invading pathogens. The innate immunity, also known as first line defense, is non-specific and responsible for the immediate action against infection. On the other hand, adaptive immunity is a highly specialized type of defense system, where the immune system first recognizes pathogen and then develops pathogen-specific defense mechanisms. In addition, adaptive immunity also generates memory cells that can handle pathogen effectively and rapidly if the system is attacked later by the same pathogen. The adaptive arm of immunity can be divided broadly into two categories; humoral and cell-mediated, responsible for activating B-cells and T-cells respectively.

Vaccination is an artificial procedure for sensitizing immune response or generating memory cells against a desired pathogen. Over the years, subunit vaccine design has become an integral part of vaccine design in which immunogenic region of protein is used instead of complete pathogen or antigen [1]. Antibodies (Abs) are one of the important components of humoral immunity where B-cells recognize antigenic regions or B-cell epitopes (BCEs) and generate antigen specific Abs. These Abs perform various functions such as phagocytosis [2], cell-mediated cytotoxicity [3], neutralization, compliment activation [4] and mast cell binding [5]. Broadly these Abs can be categorized in five classes or isolates *i.e.,* IgA, IgD, IgE, IgG, and IgM.

It has been observed in the past that particular pathogen/antigen induce defined class or subclass of Abs, for example, infections like schistosomiasis and filariasis induce a mixed response of IgE and IgG [6-8]. In case of protozoan like *Plasmodium falciparum,* Ab response of merozoite surface proteins constitutes mainly IgG1 and IgG3 subclasses [9,10]. On the other hand, viruses like rotavirus, HIV and influenza virus, are well known for inducing IgA type of response [11]. In case of IgE inducing antigens (allergens), the studies showed that the allergens have some features that make them allergenic [12]. These facts together suggest that there are desired effector functions of Abs, which are needed to encounter various types of pathogens. Thus, it is important to understand why the immune system produces different classes of antibodies against different antigens. This understanding will help an experimental biologist to design a better vaccine for the induction of systemic or mucosal immunity as well as immunotherapy. In the past, numerous databases and methods have been developed for maintaining and predicting BCEs in an antigen [13-16]. Till date, limited efforts have been made to develop the method for predicting allergens or BCEs that can induce IgE type of antibodies [17,18]. To the best of authors’ knowledge, no comprehensive attempts have been made for predicting BCEs responsible for inducing specific class of Abs or discrimination of epitopes that induce different class of Abs.

In this paper, we have made an attempt to understand the relation between amino acid sequence of epitopes and type of Abs they will induce. First we have collected IgG, IgE and IgA specific BCEs from Immune Epitope Database (IEDB). Subsequently, these three classes of epitopes were analyzed to understand which residues or group of residues are preferred among these sequences. Based on comparative analysis, we developed prediction models using various features like amino acid composition, dipeptide composition and binary profiles. We also developed a user-friendly platform for the scientific community that allows users to predict IgG, IgE and IgA specific BCEs.

## Results

### Analysis

#### Composition analysis

**Figure 1:**
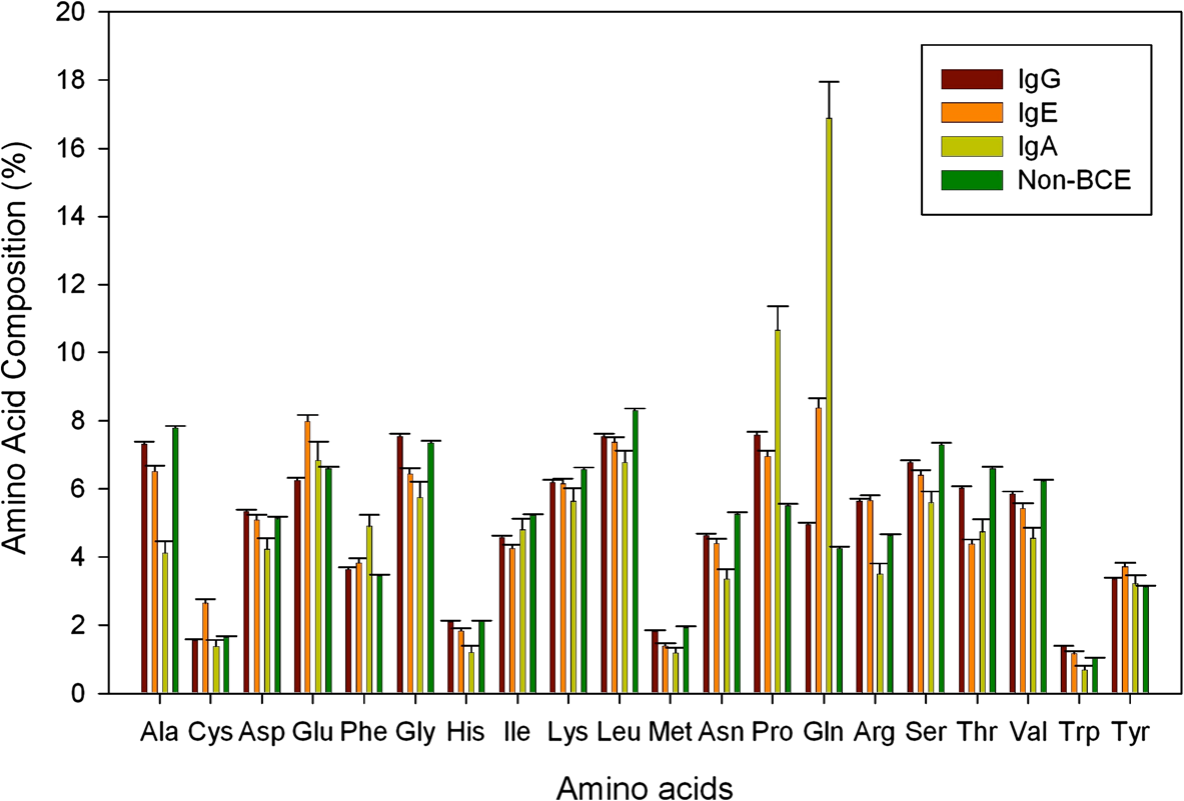
Comparison of average amino acid composition of different class of epitopes.

In order to ascertain whether certain types of residues are dominated in different classes of BCEs, the percent average amino acid composition of IgG, IgE and IgA specific BCEs and non-B-cell epitopes (non-BCEs) was calculated and compared (Figure 1). The analysis revealed that there are differences in the percent average amino acid composition profiles of four classes (IgG, IgE, IgA, and non-BCEs) of epitopes. As shown in Figure 1, certain types of residues are abundant in each class, for instance Pro and Gln are abundant in IgA inducing epitopes while Cys and Glu are found to be dominated in IgE inducing epitopes. These observations are in accordance to several previous reports, where researchers have shown that there is a propensity of Cys residues in IgE inducing epitopes, and they form stable conformational epitopes through disulphide bonds [19-25]. It has also been observed in the past that IgA binding antigenic regions are Pro/Gly rich [26,27].

Dipeptide composition provides more information than amino acid composition and has been used in the past for developing various classification models [28-30]. We have also computed and compared average dipeptide composition for each class of epitopes (IgG, IgE, IgA and non-BCE). It was observed that each class of epitopes has certain types of dipeptides having significantly higher composition (Welch’s *t*-test) than other class of epitopes. Dipeptides AS, GP, WK, YR, *etc.* are prevailing in IgG; IQ LA, NA, NE, *etc.* are frequent in IgE, and ED, FP, PF, PQ, PY, QP, *etc.* are predominant in IgA class of epitopes (Additional file 1).

#### Residue preference

**Figure 2:**
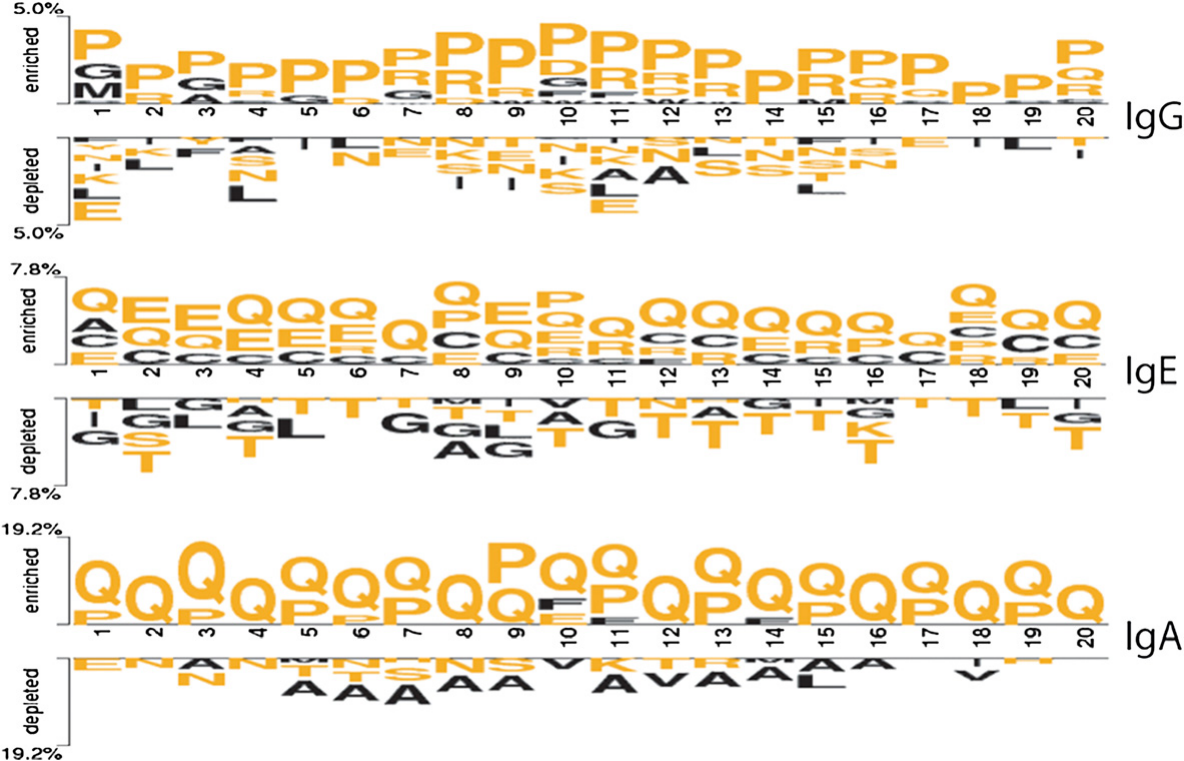
Two sample logos for each class of epitopes where epitopes of a class is taken positive and the rest of peptides as negative examples.

In order to understand the preference of residues at different positions in epitopes, we analyzed two sample logos [31] of each class (Figure 2). As shown in Figure 2, certain residues are preferred at specific positions in each class. For example, in IgG inducing epitopes, Pro, Gly and Met are preferred at the first position and Pro and Arg are preferred at second position. Overall, Pro is found to be preferred at almost every position. In IgE inducing epitopes, Gln, Glu and Cys are found to be preferred at various positions. In IgA inducing epitopes, an exclusive preference of Gln and Pro is observed (Figure 2).

#### Length of epitopes

**Figure 3:**
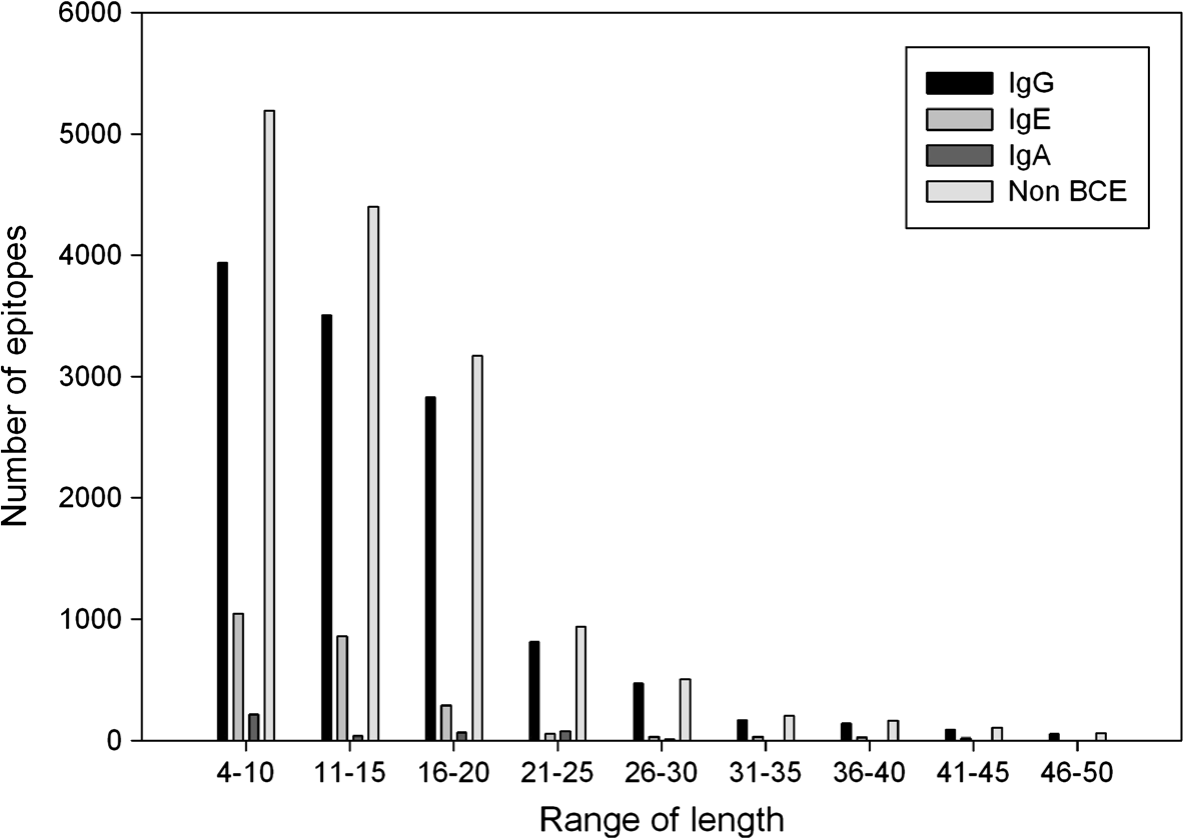
Lengthwise distributions of class-specific epitopes.

In order to understand whether the length of epitopes plays any role in inducing specific class of antibodies, we examined the length of different class of epitopes. As shown in Figure 3, most of the epitopes are between 4 and 20 residues in length, only few epitopes having length more than 40 residues. Analysis revealed that more than 55% IgA specific epitopes are less than 10 residues in length while more than 45% IgE specific epitopes are between 11 and 15 residues in length. IgA inducing epitopes have length between 4–10 residues.

#### Physico-chemical property analysis

We computed and compared the physico-chemical properties of various epitopes to understand their correlation with antibody-class specificity. We did not find any significant differences in physico-chemical properties between the classes of epitopes (Additional file 2: Figure S1), except polar, aliphatic and positively charged residues, which showed differences in their composition both in IgA and IgE classes. In the past, few attempts have been made to differentiate BCEs from non-BCEs based on physico-chemical properties and shown similar observations [32].

#### Motifs analysis

Since motif-based distinction of IgA epitopes has been reported in the past [11], we extracted motifs from other classes (IgG and IgE) considering the fact that few motifs might be present in these classes, as well. We performed MEME (see Method section) studies for the discovery of motifs in all classes and extracted 20 motifs at default parameters of MEME (Additional file 3: Table S1). We have implemented this motif information at our server where user can scan their epitopes for the presence of specific motifs.

### Models for predicting antibody-specific BCEs

In this study, we have developed numerous models for predicting IgG, IgE, and IgA specific BCEs. We built models for each class on all the datasets. The performance of models was optimized, for example, in case of SVM; parameters were tuned for all three types of kernels linear, polynomial and radial bias.

### SVM^light^ models developed on BalanceVar dataset

(i) ***Composition-based model.*** Since significant differences were observed in amino acid and dipeptide composition of each class of epitopes, first we have developed SVM models using amino acid composition as input feature and achieved maximum MCC values 0.28, 0.51 and 0.43 for IgG, IgE and IgA respectively. The performances of amino acid composition-based models are summarized in Table 1 and Additional file 3: Table S2. Next, SVM models were developed based on dipeptide composition of epitopes. Dipeptide-based models performed better than the amino acid composition-based model and achieved maximum MCC of 0.41, 0.66 and 0.44 for IgG, IgE and IgA respectively (Table 1 and Additional file 3: Table S2). Detailed performances of dipeptide-based model at different thresholds are summarized in supporting information (Additional file 3: Table S3).

(ii) ***Physico-chemical properties-based model.*** We developed models based on physico-chemical properties (PCP) using 10 physico-chemical properties of BCEs and non-BCEs. These models were further optimized in order to improve the performance. We achieved maximum MCC of 0.32, 0.29 and 0.46 for IgG, IgE and IgA respectively (Table 1 and Additional file 3: Table S2). Further, we developed models using features like composition-transition and distribution (CTD) as input, which has been used in the past for predicting BCEs [33] and achieved MCC of 0.29, 0.38 and 0.40 for IgG, IgE and IgA respectively (Table 1 and Additional file 3: Table S2). The performance of both PCP and CTD-based models on threshold dependent, as well as on threshold independent parameters were poorer than dipeptide-based models. In addition, we developed models using amino acid pairs propensity (AAP) and achieved the performance more or less similar to dipeptide-based model. AAP based model achieved maximum MCC of 0.37, 0.57 and 0.46 for IgG, IgE and IgA respectively (Table 1 and Additional file 3: Table S2).

**Table 1:** The performance of SVM models developed for predicting antibody specific BCEs on BalanceVar dataset using various features

**Input pattern**	**IgG epitope**			**IgE epitope**			**IgA epitope**		
	**ACC**	**MCC**	**AUC**	**ACC**	**MCC**	**AUC**	**ACC**	**MCC**	**AUC**
**AAC**	63.85	0.28	0.68	75.33	0.51	0.81	71.46	0.43	0.76
**AAP**	68.30	0.37	0.73	78.3	0.57	0.85	72.93	0.46	0.78
**CTD**	64.30	0.29	0.69	68.81	0.38	0.71	69.76	0.40	0.74
**DPC**	**70.42**	**0.41**	**0.76**	**82.7**	**0.66**	**0.88**	**72.07**	**0.44**	**0.78**
**PCP**	66.18	0.32	0.71	64.31	0.29	0.64	72.8	0.46	0.78

(*ACC* accuracy, *MCC* Matthew’s correlation coefficient, *AUC* area under curve).

### SVM^light^ models developed on BalanceFix dataset

(i) ***Composition-based model.*** We built models on BalanceFix dataset using amino acid composition as input feature and achieved MCC of 0.33, 0.64 and 0.39 for IgG, IgE and IgA respectively (Table 2 and Additional file 3: Table S4). Similarly, SVM models developed with dipeptide composition profile achieved MCC of 0.44, 0.70 and 0.45 for IgG, IgE and IgA respectively. The AUC values for the models were 0.77, 0.9 and 0.78 for IgG, IgE and IgA respectively. Detailed performances of dipeptide-based model at different thresholds are summarized in supporting information (Additional file 3: Table S5).

(ii) ***Physico-chemical properties-based model.*** Further, SVM models were developed using PCP which achieved MCC of 0.13, 0.16 and 0.27 for IgG, IgE and IgA respectively. The maximum performance of CTD model on this data in term of MCC was 0.15, 0.28 and 0.27 for IgG, IgE and IgA respectively (Table 2 and Additional file 3: Table S4). Furthermore, models on BalanceFix dataset using AAP achieved maximum MCC of 0.39, 0.65 and 0.49 for IgG, IgE and IgA respectively.

(iii)  ***Binary profile-based model.*** Since BalanceFix dataset consists of fixed length epitopes, therefore, we developed model using binary profile of epitopes as input features to predict antibody-specific BCEs. The binary based models achieved maximum MCC of 0.08, 0.12, 0.24 for IgG, IgE and IgA respectively (Table 2 and Additional file 3: Table S4).

**Table 2:** The performance of SVM models developed for predicting antibody specific BCEs on BalanceFix dataset using various features

**Input pattern**	**IgG epitope**			**IgE epitope**			**IgA epitope**		
	**ACC**	**MCC**	**AUC**	**ACC**	**MCC**	**AUC**	**ACC**	**MCC**	**AUC**
**AAC**	66.27	0.33	0.70	81.78	0.64	0.86	69.29	0.39	0.75
**AAP**	69.29	0.39	0.75	82.39	0.65	0.89	74.34	0.49	0.79
**CTD**	57.41	0.15	0.61	63.99	0.28	0.70	63.3	0.27	0.67
**DPC**	**71.73**	**0.44**	**0.77**	**84.96**	**0.70**	**0.90**	**72.28**	**0.45**	**0.78**
**PCP**	56.57	0.13	0.59	58.11	0.16	0.62	63.3	0.27	0.69
**BIN**	54.02	0.08	0.55	56.17	0.12	0.59	62.17	0.24	0.67

(*ACC* accuracy, *MCC* Matthew’s correlation coefficient, *AUC* Area under curve).

In addition, we also developed SVM models for each class on realistic datasets *i.e.* RealVar and RealFix datasets. The dipeptide-based SVM models performed best among the rest of the models. The performance of models developed on realistic datasets is summarized in supporting information (Additional file 3: Table S6 and Additional file 3: TableS7).

### Models developed using WEKA

We developed models based on BayesNet, Complement NaiveBayes, NaiveBayes, NaiveBayes Multinomial, SMO, IBk (kNN), J48, and RandomForest using WEKA for predicting antibody-specific BCEs. After tuning different parameters, we found that out of many algorithms of WEKA, three algorithms SMO, kNN and Random Forest performed comparatively better as shown in supporting information (Additional file 3: Table S8 and Additional file 3: TableS9). Balanced set of patterns for both variable (BalanceVar) and fixed (BalanceFix) length were used for all three classes and evaluated using five-fold cross validation technique as shown in the supplementary information (Additional file 3: Table S8 and Additional file 3: Table S9).

The best classifiers of WEKA achieved maximum accuracy 70.07% for IgG [KNN: 0, window size: 0, algorithm: LinearNN search], 81.50% for IgE [SMO, polykernel -C 250007 -O 1.0, c: 1.0, epsilon: 1.0E-12] and 71.16% for IgA [Randomforest, numFeature: 15, numTrees: 10, Seed: 1]. These results were comparable to SVM models implemented using SVM^light^, where it achieved maximum accuracy of 70.42%, 82.70% and 72.07% for IgG, IgE and IgA respectively.

### Performance of model on independent dataset

In order to evaluate the performance of our models on independent data, we first trained our models on 80% of data by ten-fold cross validation and later the performance of the best models were evaluated on remaining 20% independent data. We evaluated dipeptide based model on all the datasets for all classes of epitopes. The performances of these models are summarized in Table 3. On BalanceVar data, model (ten-fold cross validation) developed on training data achieved maximum MCC of 0.42, 0.61 and 0.39 while MCC of 0.37, 0.63 and 0.49 were achieved on evaluation datasets of IgG, IgE and IgA classes respectively. Similarly, for BalanceFix data, model performed reasonably well and achieved maximum maximum MCC of 0.42, 0.70 and 0.46 on training data while MCC of 0.43, 0.62 and 0.33 were achieved on evaluation datasets of IgG, IgE and IgA classes respectively. The performance of every ten fold cross validation model on the evaluation set was comparable to that of five fold cross validation model made on main data. Taken all these results together, it can be speculated that our model performed consistently well, and high accuracy is not due to over optimization.

**Table 3:** **The performance of dipeptide composition based SVM models, evaluated using ten-fold cross validation on training data (80**%**) and independent validation on independent data (20**%**) on BalanceEval (BalanceFix & BalanceVar) dataset**

**Dataset**		**Mode**	**Data size**	**ACC**	**MCC**	**AUC**
**BalanceVar**	IgG	Training	6063	70.88	0.42	0.76
		Evaluation	1519	68.24	0.37	0.74
	IgE	Training	1873	80.53	0.61	0.87
		Evaluation	468	81.49	0.63	0.88
	IgA	Training	322	69.60	0.39	0.75
		Evaluation	80	74.69	0.49	0.79
**BalanceFix**	IgG	Training	4893	70.87	0.42	0.76
		Evaluation	1223	71.67	0.43	0.78
	IgE	Training	1524	85.04	0.70	0.90
		Evaluation	381	80.97	0.62	0.86
	IgA	Training	213	73	0.46	0.80
		Evaluation	54	66.67	0.33	0.72

### Implementation and utility of IgPred

**Figure 4:**
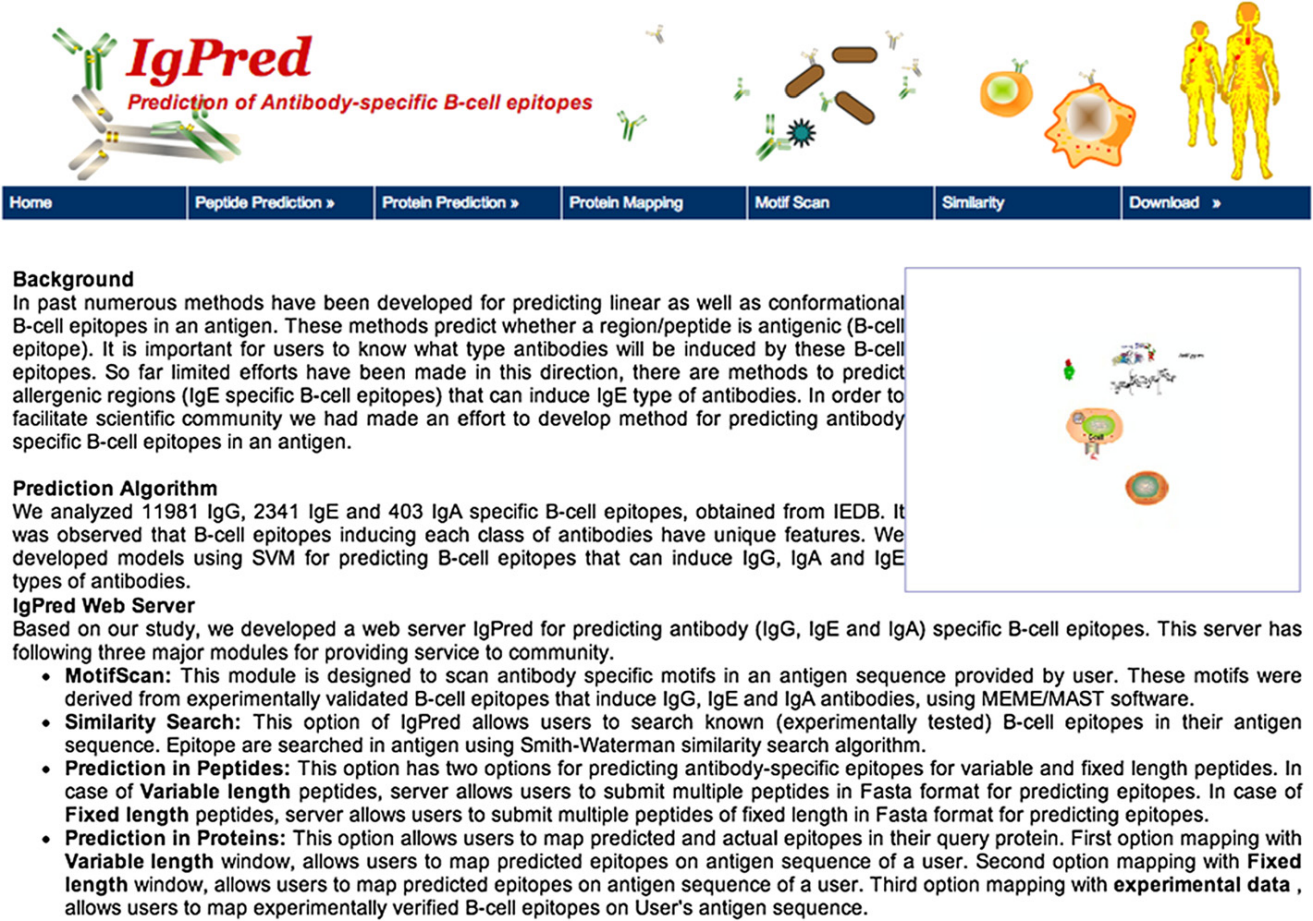
Schematic representation of IgPred webserver.

We have developed a user-friendly web server ‘IgPred’ (Figure 4) for predicting antibody-specific BCEs. A number of useful tools have been integrated to IgPred, and their descriptions is as follows:

(i)  ***Epitopes in peptides:*** This tool allows users to predict antibody-specific epitopes in their peptide sequences. User can select either variable length, or fixed length peptides option provided at server. In case of variable length peptides, the models were built on variable length data. Here, server allows users to submit multiple peptides in FASTA format for predicting epitopes. In case of fixed length peptides, the models were developed on fixed length data, so on server users may submit multiple peptides of fixed length in FASTA or plain format for predicting epitopes.

(ii)  ***Epitopes in proteins:*** This tool allows users to identify antigenic regions (BCEs) in protein sequences, which can induce particular class of Ab. There are two options for users as described above. First option is mapping with variable length, and other is mapping with fixed length window.

(iii)  ***Mapping with experimental data:*** This tool allows users to map experimentally verified class-specific BCEs on user’s antigen sequence.

(iv) ***MotifScan:*** This module is designed to scan Ab class-specific motifs in an antigen sequence provided by the users. These motifs were derived from experimentally validated BCEs that induce IgG, IgE and IgA Abs using MEME/MAST software as mentioned in method.

(v) ***Similarity search:*** This option allows users to search known (experimentally validated) BCEs in their antigen sequence using Smith-Waterman similarity search algorithm.

In the prediction tools, users can select an SVM threshold for the class of antibody that is going to be generated. We suggest that if high confidence in prediction is needed, user should select high threshold value, but at the same time sensitivity of the prediction will be compromised. The results can be downloaded immediately or can be delivered by email. The common gateway interface (CGI) script for IgPred was written using PERL 5.03. IgPred is freely available at http://crdd.osdd.net/raghava/igpred/.

## Discussion

In the past, several methods have been developed for predicting BCEs in an antigen/protein sequence from their primary structure [15,34-37]. To the best of authors’ knowledge no comprehensive method has been developed so far for predicting class–specific BCEs, except few methods developed for IgE inducing allergenic epitopes [17,38,39]. The present study is an attempt in the direction to understand differences between the BCEs that induce different classes of antibodies like IgG, IgE and IgA. We hypothesized that induction of different classes of antibodies (*i.e.* IgG, IgE and IgA) could be determined by the sequence of an epitope. Therefore, to understand this, we first extracted BCEs that induce IgG, IgE and IgA types of antibodies from the IEDB database, and then these sequences were systematically analyzed. Amino acid and dipeptide composition analysis revealed that the composition of certain residues/dipeptides is higher in certain antibody-specific epitopes than the others. In addition, few residues are preferred in a particular class suggesting that these residues or dipeptides may play an important role in class switching. For example, Pro and Gln are significantly dominant in IgA epitopes. Though Pro is usually not a preferred residue in any of the regular secondary structures, this residue might be contributing in induction of IgA. Based on these observations, it is clear that the composition of sequence (residue or dipeptide) can be used to discriminate epitopes of different class. Therefore, we developed models using amino acid and dipeptide composition as input features. As shown in results section, models based on dipeptide were able to classify the epitope of different classes with reasonably high accuracy. All models were evaluated using both threshold dependent and independent parameters. In addition to SVM models, we also developed models using various modules of WEKA package. It was observed that models based on classifier SMO, kNN and Random Forest perform better than other classifiers of WEKA. It was also observed that overall SVM-based models implemented using SVM^light^ performed better than models developed using WEKA as explained in results section.

We further developed SVM model using binary profiles of patterns as input features. In addition, we also developed models using various other features like PCP, CTD and AAP, but none of the methods achieved accuracy higher than dipeptide-based models. The performance of dipeptide-based model were also evaluated by ten-fold cross validation and performance was almost similar to five-fold. We evaluated the performance of ten-fold cross validation models on independent datasets and achieved reasonable accuracy (as mentioned in the result section). Our results suggest that performance of our models is not due to the over optimization; thus our method will be useful and effective in real life.

In our study, we used one vs. rest approach for creating datasets. It means for developing models for predicting IgA epitopes; we used IgA epitopes as positive set and the rest of the epitopes (IgE, IgG and non-BCE) as negative set. However, we have not used one vs rest approach for prediction, it means our predictions are not exclusive prediction for a single class and our models may predict a peptide inducing for more than one class of antibodies based on prediction score. In a situation where a peptide has equal score for two models then it can be assigned to both the classes if the score for both classes is more than the threshold. The dimensions provided in IgPred webserver enable users to determine the potency of any antigen to induce systemic, allergic or mucosal Ab immune response beforehand.

## Conclusion

In the present study, we have made an attempt to establish a relation between an antigenic amino acid sequence and its tendencies to generate systemic (IgG), allergic (IgE) and mucosal (IgA) type of Ab response. For the first time, *in silico* models have been developed for predicting class-specific BCEs. We have implemented our methods in the form of a web server -IgPred. We anticipate that IgPred will be beneficial in designing a better vaccine and immunotherapy, with most appropriate effector function, and several other clinical applications.

## Methods

### Dataset creation

**Figure 5:**
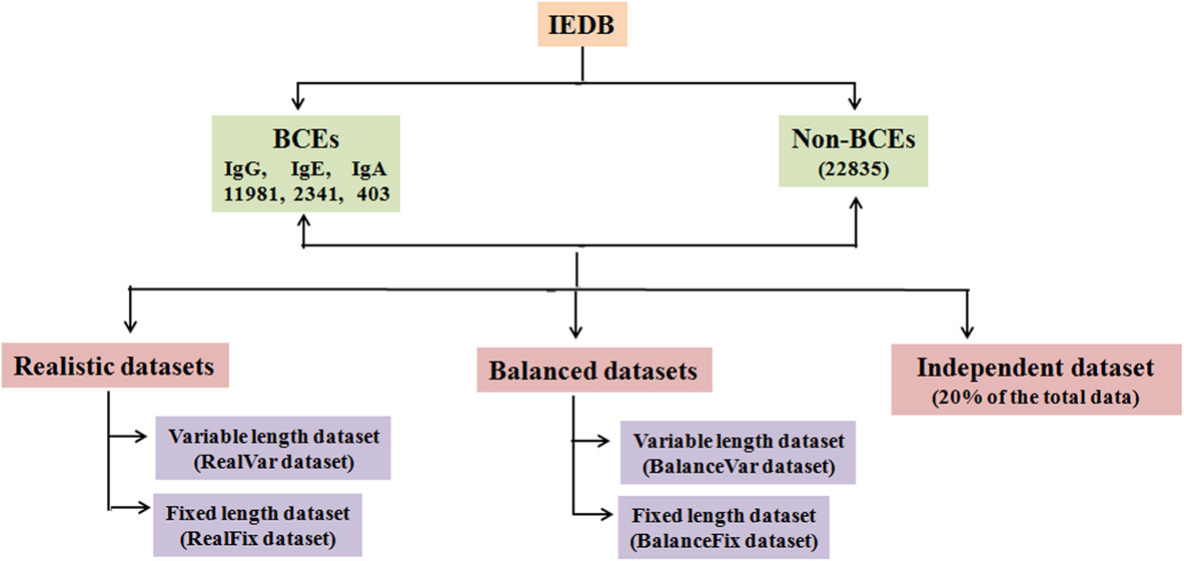
Overview of dataset creation.

In this study, datasets were derived from B-cell assays of human and mouse, which were extracted from Immune Epitope Database (IEDB) (http://www.iedb.org/). Of these sequences, B-cell assay positive epitopes were considered as BCEs (positive examples) and B-cell negative epitopes were considered as non-BCEs (negative examples). Since these sequences are of variable length (from 4–100 amino acids), only non- redundant (unique) sequences having a length between 4 to 50 amino acids were taken. These epitopes were divided into three classes on the basis of antibody heavy chain (IgG, IgE and IgA) reported in the IEDB database. Finally, we obtained 11981, 2341, 403 and 22835 sequences for IgG, IgE, IgA specific BCEs and non-BCEs respectively. Non-BCEs are entirely different from the IgG, IgE and IgA specific BCEs and nowhere present in positives. Therefore, we treated it as a separate class. From the above data, we have derived following datasets for developing various models. The approach for developing datasets (BCEs and non-BCEs) and selecting features has been adopted from the previous study by our group [35]. In this study, authors developed a method, which efficiently classify BCEs from non-BCEs. Here, in the present study, we wanted to take it to the next step to classify BCEs into class-specific BCEs. The overview of dataset creation is summarized in Figure 5.

(i)  ***Realistic dataset with variable length (RealVar dataset):*** This dataset consists of total 14725 BCEs (*i.e.,* 11981 IgG-specific, 2341 IgE-specific, 403 IgA-specific BCEs) and 22835 non-BCEs of variable lengths (Table 4)

(ii)  ***Balanced dataset with variable length (BalanceVar dataset):*** This dataset was created from the above mentioned RealVar dataset. In this dataset, positive examples were balanced by taking equal number of negative examples from total negatives. First, to avoid biases, 4942 negative examples were picked randomly from non-BCEs (22835), which is equivalent to one third of all BCEs (IgG, IgE, and IgA). Subsequently, balanced dataset of each class was developed taking equal number of negative examples randomly from total negatives (Table 4). Since machine learning technique need fixed length pattern and also it is difficult to predict epitopes in an antigen using model trained on variable length datasets as length is not fixed for scanning, we have developed two other datasets of fixed length. These datasets are derived from the above two datasets.

(iii)  ***Realistic dataset with fixed length (RealFix dataset):*** In order to utilize the full potential of machine learning techniques, we fixed the length to 20 residues as most epitopes have length up to 20 residues. This has been implemented using extension truncation technique as described previously [40,41]. After removing redundant epitopes, we got 9660, 1905, 267 and 20589 for IgG, IgE, IgA and non-BCEs respectively (Table 4).

(iv)  ***Balanced dataset with fixed length epitopes (BalanceFix dataset):*** This dataset was generated from RealFix dataset using the same process as described above for creating BalanceVar dataset. It consists of 9660, 1905, and 267 BCEs for IgG, IgE, and IgA respectively and an equal number of negative examples for the respective class as summarized in Table 4.

(v) ***Independent dataset:*** In order to create an independent dataset, 20% sequences were randomly picked from BalanceFix (for fixed length) and BalanceVar (for variable length) dataset and used as independent dataset. Model was trained on rest 80% sequences using ten-fold cross validation and then performance of the optimized model was validated on independent dataset.

**Table 4:** Various datasets used for developing prediction models in the present study

**Dataset type**	**IgG**		**IgE**		**IgA**	
	**Positive**	**Negative**	**Positive**	**Negative**	**Positive**	**Negative**
**RealVar**	11981	25579	2341	35219	403	37157
**BalanceVar**	7598	7598	2341	2341	403	403
**RealFix**	9660	22761	1905	30516	267	32154
**BalanceFix**	6116	6116	1905	1905	267	267

### One-vs-rest approach for datasets

We developed models for predicting epitopes inducing different Abs. In order to develop model for epitopes inducing a specific class of Ab (*e.g.* IgA inducing epitopes), we used IgA inducing epitopes as positive examples, and the rest of the epitopes belong to other classes (*e.g.,* IgE, IgG and non-BCE) as negative examples (Additional file 2: Figure S2). Similarly, for developing model for IgG inducing BCEs, IgG-specific BCEs were considered as positive examples and the rest of the epitopes from other classes (IgE specific, IgA specific BCEs and non-BCEs) were considered as negative examples (Additional file 2: Figure S2).

### Amino acid composition

Amino acid composition is one of the simplest features, which have been used in the past to encapsulate the global information of protein into a fixed length pattern [42,43]. Amino acid composition of a peptide is proportional to frequency of each type of residue in a peptide. As there are 20 types of residues, so there are 20 types of composition for each peptide. Thus, a vector of 20 is used to represent an epitope as described in previous studies [44].

### Dipeptide composition

Dipeptide composition is another important feature, which is used to transform the variable length of peptides to fixed length feature vectors. Dipeptide compositions have been used in earlier studies to classify various classes of peptides [44,45]. Dipeptide composition provides information of the pair of residues in contrast to a single residue and provides a fixed pattern length of 400. We computed percent of occurrence of each type of dipeptide and used this information for developing machine learning models. Following formula has been used for computing dipeptide composition of each epitope

(1) $$\text{Percentage}\;\text{of}\;\text{Dip}\left(i\right)=\frac{\text{Total}\;\text{number}\;\text{of}\;\text{Dip}\left(i\right)}{\text{Total}\;\text{number}\;\text{of}\;\text{all}\;\text{possible}\;\text{Dips}}\times 100$$

where Dip*(i)* is a dipeptide *i* out of 400 dipeptides.

To analyse the differences between dipeptide composition among all classes of epitopes (IgG-, IgE-, IgA-inducing and Non-BCE), we performed significance test (Welch’s *t*-test). For example, we first calculated average of dipeptide compositions (400 dipeptides) for both IgG(+) data and IgG(−) data. Next, we calculated the difference of means in both the datasets for each of the 400 dipeptides for IgG-, IgE-, IgA-inducing BCEs and non-BCEs respectively. We also computed whether the difference in the mean of dipeptide composition is significant or not using *t*-test. Several dipeptides show a significant difference in composition between positive and negative dataset of each class (Additional file 1).

### Binary profile of patterns

We used binary profiles of patterns for fixed length datasets where each amino acid was presented by a vector of dimension 20 as described previously [44]. Since the length of epitopes was 20, a pattern of window length 20 was represented by a vector of dimension (20 × 20). In the past, binary profile has been used for developing prediction models [44-47].

### Physico-chemical properties

It is well known that function of a peptide is governed by its primary sequence and their physico-chemical properties. Therefore, in the present study, we have selected ten commonly used physico-chemical properties. These properties include hydrophobicity, bulky side chain, net-hydrogen, stearic hindrance, amphipathicity, hydrophilicity, charge, pI value, *etc.*[48,49].

### MEME/MAST

MEME/MAST module has been used previously for discovering novel motifs in various classes of proteins and peptides [44]. We have adopted the similar strategy in the present study for discovering various motifs in epitopes using MEME/MAST module. This module comprises of two programs: one is MEME, which is used to discover motifs, and other is MAST, used for searching motifs [50,51]. In the present study, we have used MEME for discovering motifs in IgG, IgE and IgA inducing epitopes and subsequently, these discovered motifs were used further for identifying epitopes using MAST.

### Composition-transition-distribution (CTD)

CTD has been used in several machine learning algorithms [33] to get a fixed length vector from variable length data. Each peptide sequence was mapped into a string defined by three symbols. These symbols were resulted from grouping of all amino acids into three groups, on the basis of certain physico-chemical properties. For every physico-chemical property, we got a string of 1, 2 and 3 symbols; three feature given by composition, three feature given by the percent frequency of i followed by j or j followed by i (transition) and five features per symbol. Thus total 15 features representing the fractions of the entire sequence where the first, 25, 50, 75, and 100% of the candidate symbol are contained in string (distribution).

### Amino acid pairs propensity scale

It has been reported that some amino acid pairs found more frequently in BCEs than in non-BCEs. Keeping this in mind, we developed an AAP propensity scale as reported previously [33,34]. The frequencies were calculated from positive and negative data sequences [52]. AAP features can be viewed as dipeptide composition features weighted by the amino acid propensity of each dipeptide. The final vector size in AAP is 400 .

### Software for extraction of features and implementing machine-learning techniques

The calculations for different features have been carried out using in-house PERL scripts and R package (2.10.1) scripts. Plots were made using SigmaPlot 10.0. We used SVM_Light software (http://svmlight.joachims.org/) for developing SVM based models. SVM is freely available for academic use and has been used in number of research papers [29,53,54]. We also used WEKA 3.2 package (http://www.cs.waikato.ac.nz/ml/WEKA/) to develop various types of models. We used nine algorithms of WEKA package namely BayesNet, Complement NaiveBayes, NaiveBayes, NaiveBayes Multinomial, SMO, IBk, J48, and RandomForest [55].

### Evaluating the performance of models

In the present study, we used five-fold cross validation technique to evaluate the performance of our models developed for predicting antibody-specific epitopes. In this technique, one fifth of total data is used for testing and remaining data is used for training the model and this process is repeated till all instances are evaluated. Similarly, we also run ten-fold cross validation, which was preceded by evaluation on independent dataset to validate the performance of the model. In addition, in order to evaluate the performance of models, we included both threshold dependent and threshold independent parameters. In case of threshold dependent parameters, we used standard parameters like sensitivity (Sen), specificity (Spe), Overall accuracy (Acc) and Matthews’s correlation coefficient (MCC) using following equations.

(2) $$\text{Sens}=\frac{\text{TP}}{\text{TP}+\text{FN}}\times 100$$

(3) $$\text{Spec}=\frac{\text{TN}}{\text{TN}+\text{FP}}\times 100$$

(4) $$\text{Acc}=\frac{\text{TP}+\text{TN}}{\text{TP}+\text{FP}+\text{TN}+\text{FN}}\times 100$$

(5) $$\text{MCC}=\frac{\left(\text{TP}\right)\left(\text{TN}\right)-\left(\text{FP}\right)\left(\mathrm{FN}\right)}{\sqrt{\left[\text{TP}+\text{FP}\right]\left[\text{TP}+\text{FN}\right]}\left[\text{TN}+\text{FP}\right]\left[\text{TN}+\text{FN}\right]}$$

[TP = true positive; FN = false negative; TN = true negative; FP = false positive; Sens = Sensitivity; Spec = Specificity; Acc = Accuracy]

To evaluate the performance of models using threshold independent parameters, AUC (Area under curve curve) have been calculated with the help of PERF software.

Response and revision of the manuscript in light of the reviewer comments:

### Reviewers’ comments on the original manuscript

We are grateful to the reviewers’ for their useful thoughts and suggestions. We have incorporated all the suggestions of the reviewers in our manuscript. Here, we are addressing all comments of reviewer’s point-by-point.

### Reviewer number 1: Dr. M Michael Gromiha

In this work, authors have developed a method for predicting different types of B-cell epitopes. They have utilized several features such as amino acid and dipeptide compositions, physicochemical properties and binary profiles. The method showed a correlation coefficient in the range of 0.44 to 0.70 to various types of epitopes. Further, a web server has been developed for application purposes.

**Reviewer comments:** The composition analysis shows the preference of Pro in IgA. Pro is usually not a preferred residue in any of the regular secondary structures. The higher occurrence of Pro may be discussed. Further, the abundance of Cys in IgE may be commented.

**Authors’ response:** It has been shown in previous studies [56,57] that most of residues in BCEs fall in non-regular (coil or tight-turns) secondary structure. In revised paper, we have discussed the preference of Pro and Cys in IgA- and IgE-inducing epitopes respectively.

**Reviewer comments:** The residue pair preference showed the dominance of Pro with other residues only in IgG and IgA and not in IgE. This may be discussed.

**Authors’ response:** This is the first study, where types of residues preferred in the different classes of BCEs have been calculated. We have no idea (biological significance of observation) why Pro with other residues (pair) is more abundant in IgG- and IgA- inducing epitopes and not in IgE-inducing epitopes. This is an interesting point to be studied in the future for understanding the above observation.

**Reviewer comments:** It has been shown that IgE can be predicted with higher accuracy than other epitopes. The reason may be explained.

**Authors’ response:** It has been observed that IgE-inducing epitopes are more conserved in comparison to other classes of epitopes, which could be responsible for higher accuracy.

**Reviewer comments:** The expansions for the parameters used in SMO may be given.

**Authors’ response:** As suggested by the reviewer, in revised version of the manuscript, we have described SMO parameters in detail.

**Reviewer comments:** It is necessary to give the procedure used to remove the redundancy.

**Authors’ response:** In revised manuscript, the procedure to remove the redundant or duplicate peptides has been described.

**Reviewer comments:** Quality of written English: Acceptable

### Reviewer number 2: Dr Christopher Langmead (nominated by Dr Robert Murphy)

The manuscript presents an SVM-based method for predicting antibody-specific epitopes. Three classes were considered: IgA, IgE, IgG. Features included AA composition, dipeptide composition, and physio-chemical properties. Training data were obtained from the IEDB, and machine-learning methods were performed using either SVM^Light^ or WEKA. A website for performing a variety of tasks associated with epitope prediction is also reported.

The study has some flaws that need to be addressed.

**Reviewer comments:** The results obtained with SVM^Light^ are the result of tuning parameters, whereas the results obtained for the strawman models are the result of using WEKA’s default parameters. To be fair, the authors must do parameter searches for these.

**Authors’ response:** We agree with the reviewer, in our revised manuscript, we have reported the performances of strawman models after parameter optimization (see Additional file 3: Table S1).

**Reviewer comments:** Did the authors try a string kernel for the SVM^Light^ experiments? It would seem appropriate, given the nature of the data.

**Authors’ Response:** In the present study, we have tried only three kernels of SVM^Light^ namely Linear, Polynomial and Radial basis. It is possible that string kernel may perform better than above kernels, but unfortunately, our group does not have expertise in optimization of string kernel parameters. In addition, previous studies have indicated that radial basis is an efficient kernel for discriminating various types of peptide/epitopes.

**Reviewer comments:** The authors need to explain what they do in the event of a tie in their one-vs-rest approach to multi-class classification.

**Authors’ response:** In this study, we used one verses rest approach for creating datasets only, and not for predicting epitopes, for example, to develop prediction model for IgA-inducing epitopes, we created a dataset containing IgA-inducing epitopes as positive examples and remaining epitopes (IgE-, IgG-inducing epitopes and non-BCEs) as negative examples. We computed performance of models based on the threshold, for example, in IgA model if a peptide having SVM score above the threshold then it is assigned as IgA-inducing epitope. We have not used exclusive prediction for a single class and our predictors may predict a single peptide inducing more than one class of antibodies. Thus, it does not matter if a peptide has equal score for two models, and it may be predicted in two classes if the SVM score is more than the threshold. In our revised manuscript, we have clarified this point.

**Reviewer comments:** Since the matrics for their method (MCC) are apparently worse than published methods for (antibody-neutral) epitope prediction, the authors should evaluate a two-stage classification process whereby an antibody-neutral classification is performed, and then the positive results are passed to their method. This would simplify the learning task because their method would not have to learn to distinguish non-BCEs from BCEs.

**Authors’ response:** The aim of this study is to predict antibody specific BCEs instead of BCEs. For the first time, we have developed models for predicting antibody class specific-BCEs that may induce three types of antibodies (IgA, IgE and IgG). Thus, it is not possible to compare this method with the previous methods as earlier methods have been developed for predicting BCEs only.

**Reviewer comments:** The manuscript also has some flaws that need to be addressed. Primarily, they should cite and discuss other SVM-based methods for epitope prediction. Additionally, it is not clear whether the authors understand that SMO is, in fact, an algorithm for learning SVMs. It seems strange to simply list SMO among the non-SVM algorithms.

Typos: There are a number of typos that can be identified by using a spell-checker. The authors meant to say that there are five primary isotypes at the end of the first paragraph of the Background section, not six (note: they correctly list the five classes).

**Authors’ response:** As suggested by reviewer, we have cited other SVM-based methods published earlier for epitope prediction. We have also edited the manuscript as per reviewer’s suggestion. Since there are different versions of Support Vector Machines (like SVM^light^ and SMO), we tried one from SVM^light^ and other from WEKA package (SMO). As the reviewer has advised, we have placed SMO in SVM algorithms from non-SVM algorithms.

**Reviewer comments:** Quality of written English: Needs some language corrections before being published

**Authors’ Response:** We have tried our best to improve the quality of english in revised version of the manuscript.

### Reviewer number: 3

Report form:

OK

**Reviewer comments:** Quality of written English: Needs some language corrections before being published

### Referee 3: Dr Lina Ma (nominated by Dr Zhang Zhang)

This manuscript presented a method to predict B-cell epitopes that can induce a specific class of antibody and attempted to understand the relation between primary sequence of epitopes and the class of antibodies. My comments are listed as follows.

**Reviewer comments:** It is noticed that one paper published by the authors, entitled “Improved Method for Linear B-Cell Epitope Prediction Using Antigen’s Primary Sequence”, describes the method of B-cell epitope prediction”. Is the method presented here similar with that in the published one? As the authors used non-BCEs as a negative control in both papers, I wonder what is the correlation between this manuscript and the published one and it might be better to describe it clearly or discuss any issue caused.

**Authors’ response:** As indicated by reviewer, recently our group has published a paper [35] describing a method developed for predicting linear B-cell epitopes (in revised version of this paper, we have cited and discussed our recent paper). In the past, other methods have also been developed for predicting B-cell epitopes (including our recent paper [35]). In the present paper, for the first time, we have developed a method for predicting epitopes that can induce specific class of antibodies. In the revised manuscript, we have clarified the difference between IgPred and previous methods.

**Reviewer comments:** It is highly recommended that the manuscript describe their results in a more clarified and detailed manner.

a) In Figure 1, it is obvious that Pro and Gln are abundant in IgA inducing epitopes, but I do not think that Cys and Glu are dominated in IgE inducing epitopes. In Figure 2, IgA inducing epitopes contain more LP, LQ, PF, PQ, PY, QP, QL and QQ dipeptides while the IgG inducing epitopes and IgE epitopes do not tend to show a significant difference in any dipeptide compositions among the three kinds of epitopes.

b) Some statistical methods should be used to compare the difference between epitopes, and it is better to list results with significant differences.

c) Error bars should be added in the histogram of Figures to show deviations.

**Authors’ response:** We agree with the reviewer in the points (a, b and c) and after getting valuable comments from the reviewer, we performed Welch’s *t*-test for each class of epitopes (IgG, IgE, IgA, and non-BCE). As an example, we first calculated average of dipeptide compositions (400 dipeptides) for both IgG(+) data and IgG(−) data. Further with the help of *t*-test, we calculated the difference of means of both the datasets for each of 400 dipeptides. Looking at the large number of dipeptides (400), we removed Figure 2 (showing dipeptide composition as bar graph) and provided the dipeptide composition of all the three classes with their negatives along with p-value in separate Additional file 1. With such analysis, it can be observed (in the Supplementary excel sheet) that significant dipeptides such as AS, GP, WK, YR, *etc*. are found to be dominant in IgG-inducing epitopes; IQ LA, NA, NE, *etc*. are frequent in IgE-inducing epitopes; and ED, FP, PF, PQ, PY, QP, *etc*. are prevailing in IgA-inducing epitopes. We have also discussed these observations in the revised manuscript.

As per reviewer’s suggestion, we performed Welch-s *t*-test on dipeptide composition data to look at the significant difference between positive and negative data of each class.

We have also added error bars to the amino acid composition bar graph as per reviewer’s recommendation.

d) In Figure 1, non-BCEs were used as a negative control. It is better that negative control is also used in Figure 2. Similar problems also exist in sections of “Residue preference”, “Length of epitopes”, “Physico-chemical property analysis”, “Motifs analysis”. It is better that negative control (non-BCEs) should be used consistently with Figure 1.

**Authors’ response:** As suggested by the reviewer, we have added AAC of non-BCE in Figure 1. We replaced Figure 2 with excel sheet showing DPC of 400 dipeptides (Additional file 1).

e) The results of ACC, AUC, and MCC are listed in tables, what about sensitivity and specificity

**Authors’ response:** Since there were about six features for each of the three classes of epitopes, we did not include sensitivity and specificity in the result tables. After getting suggestion of the reviewer, we created additional tables (Table S2 and S4) containing sensitivity and specificity and incorporated as supplementary information (Additional file 3: Table S2 and Additional file 3: Table S4).

f) What does AAP mean? What is the difference between AAP and DPC?

**Authors’ response:** AAP (Amino acid propensity) is a feature, which is derived from DPC of the datasets. This feature has been exploited in previous B-cell epitope prediction algorithms [33,40,41,43]. This represents the DPC of a protein/amino acid sequence weighted by the amino acid propensity of each dipeptide in a matrix made by both positive and negative data. We have edited the manuscript by describing it in detail.

**Reviewer comments: Discussion Section-** At the end of the first paragraph, it is concluded that SVM performed better than WEKA. This is an important conclusion of this paper, which should be explained in detail.

**Authors’ response:** As we can observe in the supplementary tables S8 and S9, algorithms of WEKA (IBk, Random Forest and SMO) could perform reasonably well with DPC as input feature. At the same time, using SVM^light^ with DPC as input feature, the performances of different models were significantly better than those of WEKA as a whole. As suggested by the reviewer, we have discussed the performances of SVM and WEKA modules in detail in the discussion section.

**Reviewer comments:** At the end of paragraph 2, please explain “reasonable accuracy” in detail?

**Authors’ response:** As suggested by the reviewer, we have explained the accuracies of our models on independent dataset in detail. For BalanceVar data, model (ten-fold cross validation) developed on training data achieved maximum MCC of 0.42, 0.61 and 0.39 while MCC of 0.37, 0.63 and 0.49 were achieved on evaluation datasets of IgG, IgE and IgA classes respectively. Similarly, for BalanceFix data, model performed well and achieved maximum MCC of 0.42, 0.70 and 0.46 on training data while MCC of 0.43, 0.62 and 0.33 were achieved on evaluation datasets of IgG, IgE and IgA classes respectively. We have also discussed this issue in the revised manuscript as per reviewer’s suggestion.

**Reviewer comments:** IgA inducing epitopes are quite different from IgE or IgG inducing epitopes in AAC and AAP comparison. However, SVM models for predicting IgA inducing epitopes do not seem to perform better than that for predicting IgE and IgG inducing epitopes. This is really confusing for me, or it might be better to provide explanations for this result.

**Authors’ response:** We agree with the reviewer that IgA epitopes are quite different. We examined the performance of prediction of IgA inducing epitopes, as well as the reason for the poor performance of these models. It could be due to the fact that IgA inducing epitopes are very limited in comparison to non-IgA inducing epitopes. It is a well-known fact that machine learning techniques, particularly SVM-based models perform poor especially when positive and negative dataset is unbalanced. We have mentioned these points in the revised manuscript.

**Reviewer comments:** As there have been methods for predicting IgE inducing epitopes, what is the difference between previous methods and the models described in the manuscript?

**Authors’ response:** We agree with the reviewer that there are methods developed for prediction of IgE inducing epitopes (one is from our own group [17]). The current study can be considered as an extension of the previous studies. This study offers researcher to compare the potential of an epitope to induce systemic type (IgG), mucosal type (IgA) and inflammatory type (IgE) of antibody immune response. Thus in contrast to the predictors of IgE inducing epitopes, our web server is more comprehensive with reference to antibody immune response.

### Reviewers’ comments on the revised manuscript

#### Referee 3: Dr Lina Ma (nominated by Dr Zhang Zhang)

**Reviewer comments:** The authors have answered all the questions seriously, while there are still some mistakes in the revised version. It is better that the authors check the manuscript carefully before submission.

Section “Composition analysis”, paragraph 1, line 6, the word “Glu” should be Gln. Section “Discussion”, paragraph 1, lines 19–20, this sentence seems incomplete.

The authors say that they have mentioned these points in the revised manuscript in answering my third question of Section “Discussion”, while I did not find where they have mentioned.

**Authors’ response:** We are thankful to the reviewer for appreciating our efforts. As suggested by the reviewer, we have edited the manuscript and incorporated all the suggestions. The performance of WEKA has been discussed in result and discussion section after getting the comments of reviewer.

## Abbreviations

AAC: Amino acid composition; DPC: Dipeptide composition; MCC: Matthews’s correlation coefficient; CTD: Composition-transition-distribution; AAP: Amino acid pairs propensity; AUC: Area under the curve; SVM: Support vector machine.

## Competing interests

The authors declare that they have no competing interests.

## Authors’ contributions

SG and HRA created datasets and developed models. SG created the backend web server and AG contributed in the development of the front end user interface. AG and SG analyzed the data and wrote the manuscript. OSDD consortium provides discussions and feedback on this problem. GPSR conceived the project, coordinated it and refined the final manuscript drafted by AG and SG. All authors have read and approved the final manuscript.

## Supplementary Material

Additional file 1 Comparison of dipeptide composition of various types of BCEs and non-BCEs. Click here for file

Additional file 2: Figure S1 Comparison of physico-chemical properties of various types of BCEs and non-B-cell-epitopes. **Figure S2.** One vs. rest approach used in developing datasets. Click here for file

Additional file 3: Table S1 List of top 20 motifs discovered in three classes of epitopes. **Table S2.** The performance of SVM models developed for predicting antibody specific BCEs on BalanceVar dataset. **Table S3.** The performance of dipeptide-based model on BalanceVar dataset, evaluated using five-fold cross validation technique. **Table S4.** The performance of SVM models developed for predicting antibody specific BCEs on BalanceFix dataset. **Table S5.** The performance of dipeptide-based model evaluated using five-fold cross validation, performance was evaluated on BalanceFix dataset. **Table S6.** The performance of SVM-based models developed using various features for predicting antibody specific B-cell epitopes on RealFix dataset. **Table S7.** The performance of SVM based models developed using various features for predicting antibody specific B-cell epitopes on RealVar dataset. **Table S8.** Performance of WEKA classifiers developed using various input features for different classes of epitopes on BalanceFix dataset. **Table S9.** The performance of WEKA classifiers developed using various input features for different classes of epitopes on BalanceVar dataset. Click here for file
