# Supplementary material for: Identification of B-cell epitopes in an antigen for inducing specific class of antibodies
Source: Biol Direct. 2013 Oct 30;8:27. doi: 10.1186/1745-6150-8-27 (PMC3831251; doi:10.1186/1745-6150-8-27)
Supplement: Additional file 2: Figure S1 — Comparison of physico-chemical properties of various types of BCEs and non-B-cell-epitopes. Figure S2. One vs. rest approach used in developing datasets. [file 1745-6150-8-27-S2.docx]

**Additional file 2**

**Identification of B-cell epitopes in an antigen for inducing specific class of antibodies.**

Sudheer Gupta, Hifzur Rahman Ansari, Ankur Gautam, Open Source Drug Discovery Consortium2 and Gajendra P. S. Raghava*

**Figure S1.** Comparison of physicochemical properties among three classes of epitopes along with non-B-cell epitopes.

**
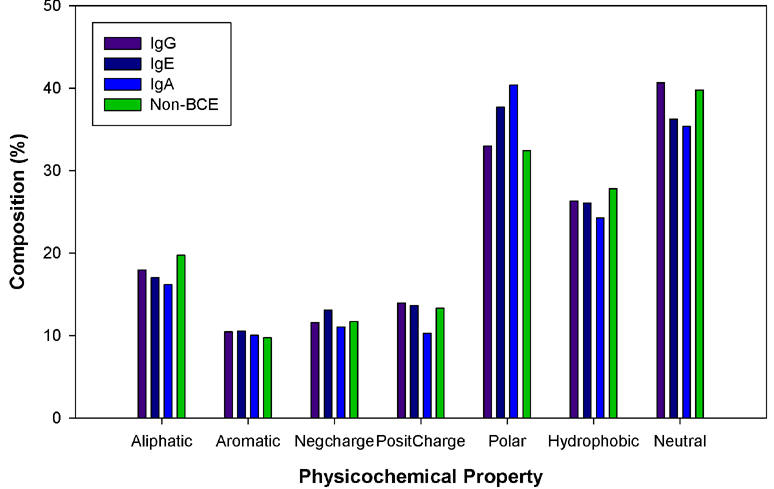
**

**Figure S2.** One vs. rest approach used in developing datasets.

**
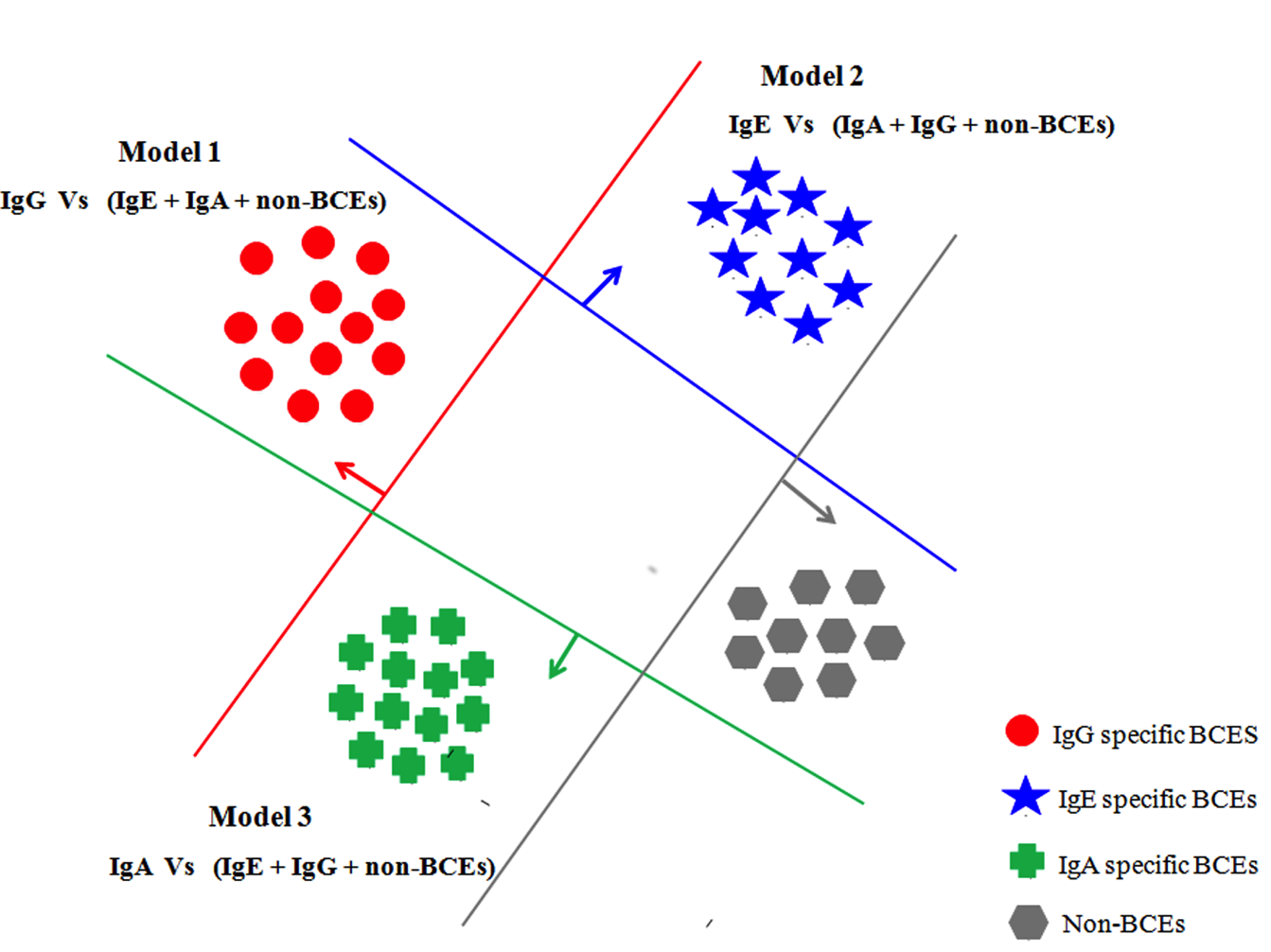
**
