# Supplementary material for: Identification of B-cell epitopes in an antigen for inducing specific class of antibodies
Source: Biol Direct. 2013 Oct 30;8:27. doi: 10.1186/1745-6150-8-27 (PMC3831251; doi:10.1186/1745-6150-8-27)
Supplement: Additional file 3: Table S1 — List of top 20 motifs discovered in three classes of epitopes. Table S2. The performance of SVM models developed for predicting antibody specific BCEs on BalanceVar dataset. Table S3. The performance of dipeptide-based model on BalanceVar dataset, evaluated using five-fold cross validation technique. Table S4. The performance of SVM models developed for predicting antibody specific BCEs on BalanceFix dataset. Table S5. The performance of dipeptide-based model evaluated using five-fold cross validation, performance was evaluated on BalanceFix dataset. Table S6. The performance of SVM-based models developed using various features for predicting antibody specific B-cell epitopes on RealFix dataset. Table S7. The performance of SVM based models developed using various features for predicting antibody specific B-cell epitopes on RealVar dataset. Table S8. Performance of WEKA classifiers developed using various input features for different classes of epitopes on BalanceFix dataset. Table S9. The performance of WEKA classifiers developed using various input features for different classes of epitopes on BalanceVar dataset. [file 1745-6150-8-27-S3.docx]

**Identification of B-cell epitopes in an antigen for inducing specific class of antibodies.**

Sudheer Gupta, Hifzur Rahman Ansari, Ankur Gautam, Open Source Drug Discovery Consortium2 and Gajendra P. S. Raghava*

**Additional file 3**

**Table S1.** List of top 20 motifs discovered by MEME studies for three classes of epitopes.

|  | **IgG epitopes** | **IgE epitopes** | **IgA epitopes** |
| --- | --- | --- | --- |
| **Motif 1** | LY[QE][EA]FDEMEEC | QQQQPQQQFPQQQQP | QPY[PQ]Q[QP]QPFPQ[QP]Q[PQ][FQY] |
| **Motif 2** | LSG[KR]PAI[IV]PDRE[VA]LY | [CN][SA]QWGW[CT]G[ST]xD | [EK]E[ED]EEDK[EK][EDR][DKE]E |
| **Motif 3** | [QFP][PF][QP]Q[PQ]QQPFPQ[QP][QP]Q[PQ] | [FW][YA][PS][EQD][LY][LMF][HR][QR][LF]Y[QH] | [HE]K[SHT][AG]I[VL]T[LV]TY[DH]SE[WET]QR[DQT][QK]F |
| **Motif 4** | G[NS]D[YW]EDRYYRENM[HYN]R | [PH][MPY]H[IPF][RHD]LS[FG] | FEEIRNLALQT |
| **Motif 5** | [MP]PPPGM[RI]PP[PM][GP] | [LI][LP][FY][VC][MR][GE][NG][ESG][AK][EV]P | CCQ[QH]LWQIPEQS[RQ]C |
| **Motif 6** | [KQ][MW][DK]AEF[RH][HR][DW]S[GS]Y[EM]VH | KC[KG][VI]NA[LP]YKIS[AG][SL]TN | G[TD]I[FC][DN][NT][FM][LH][IY]TN |
| **Motif 7** | MEEC[AS][SQ][HA]LPYIEQ[GA][MQ] | [YDP][GRPS][RQV][DE]P[YF][ST][PE]S[QP][DYS] | LCLIGCKPPIGEHWG |
| **Motif 8** | V[SA]PTHYVPESD | PE[VG]D[DL]E[AI]L[EL][KRQ][FK] | [IN][PYG][CKE][GR][KD][VN][VT]L[IQR][NQI][HKR][ND][IV][RA][CHF] |
| **Motif 9** | N[AV][DN][PG]NA[NG][PG]NA[NG][PG]NA[NG] | [RV][HR][AS][KP]A[HQ]IL[QR]W[GQ] | GG[SG][GR]G[GR]A[GR]AGG[RG]GRG |
| **Motif 10** | PYIEQGMQLA[EA][QE]FKQ | TY[DGT]N[EKD]C[LN][LF]C[AN][HAY] | VLQESTYQLVQQLCC |
| **Motif 11** | H[IV][ST]GHRMAWDMM[LM]NW | [LA][KRA]S[LV]P[QG][QK]CG[VL]R | [VQ][SP][FV][QP]Q[PL]Q[QL]Q[YN]P |
| **Motif 12** | [AT]SG[AG][SA][GA][QG][SAP]G[AT][SA]GA[SG]G | [NG][LE]A[RK]TT[PA][DQ]R[QR][AT]ACNC | GGGYVKLFPNS |
| **Motif 13** | GSSYGFQYSPG | [PV][SA]G[AT]WY[YS][VL][PA][LM][GA] | [KH]G[SD][PS][CE][TY]N[VI][AM][VF][NG]P[GD][DI]C |
| **Motif 14** | AEQFKQK[AV]LGLLQ[TR][AS] | [ILA][NDG][TKN][QDP][YF][SKLT][KDPW][YAR][PLS][SKLY][YF] | [AHNP][LC][SHY][ACRT][TS][FI][EA][PS]F[GS][INV][FKL]G |
| **Motif 15** | NPKPQRKTKRNTNRR | E[IV]V[PR]N[SAT][VAN]E[EDQ][KRE][HLY] | [DA]L[YN]CY[EW][QP]LND |
| **Motif 16** | NDIRVEESIYQ | [QA][DEK]KT[EK][INE]P[ATC] | AVYFKEQFLDGDGWT |
| **Motif 17** | H[PS][NY][IQ]EE[VA]A[LM][GSE][HLNQT][ETK][GV]E[ID]PFY[GW][KRV] | [QM][AT][MA][ES]DI[KS][QLV][ML][ED]A | RDHSYQEEAAM |
| **Motif 18** | FGKGGIVTCAMFTCK | [QR][SF][LKF]V[YA]PF[PA] | EEKDKGLQTSQDARF |
| **Motif 19** | ILIGVIITWIGMNSR | A[FY]N[HQ]FG[PEK][NGR][LA][GID]QRMPR | [MV]N[FQ][DM]I[PN]E[EA][IK]KQL[QL][QE]F |
| **Motif 20** | FLVNTWKSKK[NC]PMGF | Q[GQ][DG]RR[CH]QS | HKSDFGKFVLSSGKF |

**Table S2.** The detailed performance of SVM models developed using various features for predicting antibody specific BCEs on BalanceVar dataset.

| **Input Feature** | **IgG Epitope** | | | | | **IgE Epitope** | | | | | **IgA Epitope** | | | | |
| --- | --- | --- | --- | --- | --- | --- | --- | --- | --- | --- | --- | --- | --- | --- | --- |
|  | SEN | SPEC | ACC | MCC | AUC | SEN | SPEC | **ACC** | **MCC** | AUC | SEN | SPEC | **ACC** | **MCC** | AUC |
| **AAC** | 62.45 | 65.24 | 63.85 | 0.28 | 0.68 | 73.41 | 77.23 | 75.33 | 0.51 | 0.81 | 70.97 | 71.94 | 71.46 | 0.43 | 0.76 |
| **AAP** | 68.11 | 68.50 | 68.30 | 0.37 | 0.73 | 74.26 | 82.30 | 78.3 | 0.57 | 0.85 | 73.95 | 71.94 | 72.93 | 0.46 | 0.78 |
| **CTD** | 63.46 | 65.12 | 64.30 | 0.29 | 0.69 | 69.61 | 68.02 | 68.81 | 0.38 | 0.71 | 70.22 | 69.30 | 69.76 | 0.40 | 0.74 |
| **DPC** | **72.05** | **68.82** | **70.42** | **0.41** | **0.76** | **75.89** | **89.44** | **82.7** | **0.66** | **0.88** | **64.52** | **79.38** | **72.07** | **0.44** | **0.78** |
| **PCP** | 66.53 | 65.84 | 66.18 | 0.32 | 0.71 | 64.62 | 64.01 | 64.31 | 0.29 | 0.64 | 72.21 | 73.38 | 72.8 | 0.46 | 0.78 |

SEN: Sensitivity; SPEC: Specificity

**Table S3.** Performance of dipeptide-based model with five fold cross validation on BalanceVar dataset, for all three classes.

**IgG class**

Thres TP FP TN FN Sen Spec Accu MCC

-1 7398 6993 682 200 97.37 8.89 52.90 0.13

-0.9 7274 6594 1081 324 95.74 14.08 54.70 0.17

-0.8 7222 6122 1553 376 95.05 20.23 57.45 0.23

-0.7 7121 5664 2011 477 93.72 26.20 59.79 0.27

-0.6 6996 5120 2555 602 92.08 33.29 62.54 0.31

-0.5 6853 4641 3034 745 90.19 39.53 64.74 0.34

-0.4 6648 4140 3535 950 87.50 46.06 66.67 0.37

-0.3 6411 3667 4008 1187 84.38 52.22 68.22 0.39

-0.2 6143 3245 4430 1455 80.85 57.72 69.23 0.40

-0.1 5831 2808 4867 1767 76.74 63.41 70.05 0.41

0 5474 2393 5282 2124 72.05 68.82 70.42 0.41##

0.1 5095 2028 5647 2503 67.06 73.58 70.33 0.41

0.2 4726 1666 6009 2872 62.20 78.29 70.29 0.41

0.3 4261 1371 6304 3337 56.08 82.14 69.17 0.40

0.4 3786 1134 6541 3812 49.83 85.22 67.62 0.38

0.5 3284 916 6759 4314 43.22 88.07 65.76 0.35

0.6 2804 732 6943 4794 36.90 90.46 63.82 0.32

0.7 2349 576 7099 5249 30.92 92.50 61.86 0.30

0.8 1879 433 7242 5719 24.73 94.36 59.72 0.27

0.9 1448 303 7372 6150 19.06 96.05 57.75 0.24

1 1020 162 7513 6578 13.42 97.89 55.87 0.21

**IgE class-**

Thres TP FP TN FN Sen Spec Accu MCC

-1 2328 2131 236 15 99.36 9.97 54.44 0.21

-0.9 2292 2028 339 51 97.82 14.32 55.86 0.22

-0.8 2282 1872 495 61 97.40 20.91 58.96 0.28

-0.7 2263 1693 674 80 96.59 28.47 62.36 0.34

-0.6 2240 1530 837 103 95.60 35.36 65.33 0.39

-0.5 2210 1333 1034 133 94.32 43.68 68.87 0.44

-0.4 2160 1099 1268 183 92.19 53.57 72.78 0.50

-0.3 2113 908 1459 230 90.18 61.64 75.84 0.54

-0.2 2053 726 1641 290 87.62 69.33 78.43 0.58

-0.1 1998 562 1805 345 85.28 76.26 80.74 0.62

0 1920 429 1938 423 81.95 81.88 81.91 0.64

0.1 1842 332 2035 501 78.62 85.97 82.31 0.65

0.2 1778 250 2117 565 75.89 89.44 82.70 0.66##

0.3 1683 190 2177 660 71.83 91.97 81.95 0.65

0.4 1564 138 2229 779 66.75 94.17 80.53 0.63

0.5 1436 115 2252 907 61.29 95.14 78.30 0.60

0.6 1268 97 2270 1075 54.12 95.90 75.12 0.55

0.7 1085 81 2286 1258 46.31 96.58 71.57 0.50

0.8 877 72 2295 1466 37.43 96.96 67.35 0.43

0.9 637 61 2306 1706 27.19 97.42 62.48 0.35

1 405 34 2333 1938 17.29 98.56 58.13 0.27

**IgA class-**

Thres TP FP TN FN Sen Spec Accu MCC

-1 402 416 1 1 99.75 0.24 49.15 -0.00

-0.9 399 410 7 4 99.01 1.68 49.51 0.03

-0.8 398 402 15 5 98.76 3.60 50.37 0.08

-0.7 395 386 31 8 98.01 7.43 51.95 0.13

-0.6 383 368 49 20 95.04 11.75 52.68 0.12

-0.5 379 351 66 24 94.04 15.83 54.27 0.16

-0.4 368 317 100 35 91.32 23.98 57.07 0.21

-0.3 354 271 146 49 87.84 35.01 60.98 0.27

-0.2 339 232 185 64 84.12 44.36 63.90 0.31

-0.1 322 198 219 81 79.90 52.52 65.98 0.34

0 310 171 246 93 76.92 58.99 67.80 0.36

0.1 294 144 273 109 72.95 65.47 69.15 0.39

0.2 278 108 309 125 68.98 74.10 71.59 0.43

0.3 260 86 331 143 64.52 79.38 72.07 0.44##

0.4 241 60 357 162 59.80 85.61 72.93 0.47

0.5 221 35 382 182 54.84 91.61 73.54 0.50

0.6 195 27 390 208 48.39 93.53 71.34 0.47

0.7 177 18 399 226 43.92 95.68 70.24 0.47

0.8 152 16 401 251 37.72 96.16 67.44 0.42

0.9 114 13 404 289 28.29 96.88 63.17 0.35

1 74 6 411 329 18.36 98.56 59.15 0.29

**Table S4.** The detailed performance of SVM models developed using various features for predicting antibody specific BCEs on BalanceFix dataset.

| **Input Feature** | **IgG Epitope** | | | | | **IgE Epitope** | | | | | **IgA Epitope** | | | | |
| --- | --- | --- | --- | --- | --- | --- | --- | --- | --- | --- | --- | --- | --- | --- | --- |
|  | SEN | SPEC | ACC | MCC | AUC | SEN | SPEC | **ACC** | **MCC** | AUC | SEN | SPEC | **ACC** | **MCC** | AUC |
| **AAC** | 67.10 | 65.43 | 66.27 | 0.33 | 0.70 | 77.17 | 86.40 | 81.78 | 0.64 | 0.86 | 68.54 | 70.04 | 69.29 | 0.39 | 0.75 |
| **AAP** | 71.42 | 67.15 | 69.29 | 0.39 | 0.75 | 81.73 | 83.04 | 82.39 | 0.65 | 0.89 | 45.69 | 92.88 | 74.34 | 0.49 | 0.79 |
| **CTD** | 58.44 | 56.38 | 57.41 | 0.15 | 0.61 | 65.72 | 62.26 | 63.99 | 0.28 | 0.70 | 55.06 | 71.54 | 63.3 | 0.27 | 0.67 |
| **DPC** | **75.28** | **68.18** | **71.73** | **0.44** | **0.77** | **86.04** | **83.88** | **84.96** | **0.70** | **0.90** | **70.41** | **74.16** | **72.28** | **0.45** | **0.78** |
| **PCP** | 52.98 | 60.17 | 56.57 | 0.13 | 0.59 | 59.00 | 57.22 | 58.11 | 0.16 | 0.62 | 64.42 | 62.17 | 63.3 | 0.27 | 0.69 |

SEN: Sensitivity; SPEC: Specificity

**Table S5.** Performance of dipeptide based model with five fold cross validation on BalanceFix dataset for all three classes.

**IgG class-**

Thres TP FP TN FN Sen Spec Accu MCC

-1 5892 5208 908 224 96.34 14.85 55.59 0.19

-0.9 5716 4665 1451 400 93.46 23.72 58.59 0.24

-0.8 5644 4152 1964 472 92.28 32.11 62.20 0.31

-0.7 5558 3725 2391 558 90.88 39.09 64.99 0.35

-0.6 5451 3406 2710 665 89.13 44.31 66.72 0.37

-0.5 5350 3090 3026 766 87.48 49.48 68.48 0.40

-0.4 5208 2778 3338 908 85.15 54.58 69.87 0.42

-0.3 5040 2498 3618 1076 82.41 59.16 70.78 0.43

-0.2 4852 2215 3901 1264 79.33 63.78 71.56 0.44

-0.1 4604 1946 4170 1512 75.28 68.18 71.73 0.44##

0 4316 1695 4421 1800 70.57 72.29 71.43 0.43

0.1 4021 1456 4660 2095 65.75 76.19 70.97 0.42

0.2 3667 1234 4882 2449 59.96 79.82 69.89 0.41

0.3 3351 1026 5090 2765 54.79 83.22 69.01 0.40

0.4 3026 840 5276 3090 49.48 86.27 67.87 0.38

0.5 2683 702 5414 3433 43.87 88.52 66.20 0.36

0.6 2361 568 5548 3755 38.60 90.71 64.66 0.34

0.7 2031 454 5662 4085 33.21 92.58 62.89 0.32

0.8 1686 368 5748 4430 27.57 93.98 60.78 0.29

0.9 1293 278 5838 4823 21.14 95.45 58.30 0.25

1 885 136 5980 5231 14.47 97.78 56.12 0.22

**IgE class-**

Thres TP FP TN FN Sen Spec Accu MCC

-1 1886 1618 287 19 99.00 15.07 57.03 0.26

-0.9 1864 1502 403 41 97.85 21.15 59.50 0.30

-0.8 1855 1362 543 50 97.38 28.50 62.94 0.36

-0.7 1841 1219 686 64 96.64 36.01 66.33 0.41

-0.6 1823 1068 837 82 95.70 43.94 69.82 0.46

-0.5 1801 932 973 104 94.54 51.08 72.81 0.51

-0.4 1775 777 1128 130 93.18 59.21 76.19 0.56

-0.3 1740 640 1265 165 91.34 66.40 78.87 0.60

-0.2 1711 518 1387 194 89.82 72.81 81.31 0.64

-0.1 1672 410 1495 233 87.77 78.48 83.12 0.67

0 1639 307 1598 266 86.04 83.88 84.96 0.70##

0.1 1595 234 1671 310 83.73 87.72 85.72 0.72

0.2 1557 183 1722 348 81.73 90.39 86.06 0.72

0.3 1506 154 1751 399 79.06 91.92 85.49 0.72

0.4 1457 131 1774 448 76.48 93.12 84.80 0.71

0.5 1391 110 1795 514 73.02 94.23 83.62 0.69

0.6 1304 99 1806 601 68.45 94.80 81.63 0.66

0.7 1199 87 1818 706 62.94 95.43 79.19 0.62

0.8 1029 78 1827 876 54.02 95.91 74.96 0.55

0.9 809 72 1833 1096 42.47 96.22 69.34 0.46

1 563 41 1864 1342 29.55 97.85 63.70 0.38

**IgA class-**

Thres TP FP TN FN Sen Spec Accu MCC

-1 259 245 22 8 97.00 8.24 52.62 0.11

-0.9 253 233 34 14 94.76 12.73 53.75 0.13

-0.8 248 221 46 19 92.88 17.23 55.06 0.15

-0.7 245 213 54 22 91.76 20.22 55.99 0.17

-0.6 239 199 68 28 89.51 25.47 57.49 0.20

-0.5 234 184 83 33 87.64 31.09 59.36 0.23

-0.4 224 164 103 43 83.90 38.58 61.24 0.25

-0.3 216 130 137 51 80.90 51.31 66.10 0.34

-0.2 207 105 162 60 77.53 60.67 69.10 0.39

-0.1 193 92 175 74 72.28 65.54 68.91 0.38

0 188 69 198 79 70.41 74.16 72.28 0.45##

0.1 178 53 214 89 66.67 80.15 73.41 0.47

0.2 162 40 227 105 60.67 85.02 72.85 0.47

0.3 152 25 242 115 56.93 90.64 73.78 0.51

0.4 139 22 245 128 52.06 91.76 71.91 0.48

0.5 130 16 251 137 48.69 94.01 71.35 0.48

0.6 127 13 254 140 47.57 95.13 71.35 0.49

0.7 117 8 259 150 43.82 97.00 70.41 0.48

0.8 104 6 261 163 38.95 97.75 68.35 0.45

0.9 90 2 265 177 33.71 99.25 66.48 0.44

1 65 1 266 202 24.34 99.63 61.99 0.36

**Table S6.** The performance of SVM-based models developed using various features for predicting antibody specific B-cell epitopes on RealFix dataset.

| **Class of Antibodies** | **Feature** | **Sen** | **Spec** | **Acc** | **MCC** | **AUC** |
| --- | --- | --- | --- | --- | --- | --- |
| **IgG** | AAC | 69.49 | 71.89 | 71.18 | 0.39 | 0.75 |
|  | AAP | 79.82 | 69.76 | 72.76 | 0.46 | 0.79 |
|  | CTD | 37.03 | 85.17 | 70.83 | 0.25 | 0.67 |
|  | DPC | **74** | **78.74** | **77.33** | **0.5** | **0.83** |
|  | PCP | 15.82 | 93.71 | 70.5 | 0.15 | 0.61 |
|  | BIN | 61.45 | 52.01 | 54.82 | 0.12 | 0.60 |
| **IgE** | AAC | 72.91 | 88.12 | 87.23 | 0.4 | 0.84 |
|  | AAP | 80.16 | 89.46 | 88.92 | 0.46 | 0.89 |
|  | CTD | 19.27 | 98.01 | 93.38 | 0.24 | 0.74 |
|  | DPC | **77.38** | **94** | **93.02** | **0.55** | **0.9** |
|  | PCP | 3.88 | 99.61 | 93.99 | 0.11 | 0.68 |
|  | BIN | 10.76 | 97.28 | 92.20 | 0.11 | 0.60 |
| **IgA** | AAC | 41.2 | 98.85 | 98.37 | 0.3 | 0.77 |
|  | AAP | 40.07 | 99.13 | 98.64 | 0.33 | 0.75 |
|  | CTD | 12.36 | 99.93 | 99.21 | 0.27 | 0.71 |
|  | DPC | **40.82** | **99.67** | **99.18** | **0.45** | **0.76** |
|  | PCP | 3 | 99.98 | 99.18 | 0.13 | 0.67 |
|  | BIN | 13.86 | 99.57 | 98.87 | 0.17 | 0.67 |

Sen: Sensitivity; Spec: Specificity

**Table S7.** The performance of SVM based models developed using various features for predicting antibody specific B-cell epitopes on RealVar dataset.

| **Class of Antibodies** | **Feature** | **Sen** | **Spec** | **Acc** | **MCC** | **AUC** |
| --- | --- | --- | --- | --- | --- | --- |
| **IgG** | AAC | 44.9 | 84.36 | 71.77 | 0.32 | 0.7 |
|  | **AAP** | **55.30** | **84.41** | **75.74** | **0.41** | **0.79** |
|  | CTD | 25.04 | 92.9 | 72.68 | 0.25 | 0.67 |
|  | DPC | 56.58 | 82.19 | 74.02 | 0.39 | 0.73 |
|  | PCP | 22.94 | 89.17 | 69.44 | 0.16 | 0.61 |
| **IgE** |  |  |  |  |  |  |
|  | AAC | 42.93 | 94.27 | 91.07 | 0.33 | 0.7 |
|  | **AAP** | **52.81** | **97.12** | **94.51** | **0.5** | **0.89** |
|  | CTD | 8.82 | 99.36 | 94.04 | 0.18 | 0.74 |
|  | DPC | 54.76 | 95.91 | 93.35 | 0.47 | 0.86 |
|  | PCP | 40.21 | 83.67 | 81.11 | 0.15 | 0.68 |
| **IgA** |  |  |  |  |  |  |
|  | AAC | 34.99 | 99.43 | 98.74 | 0.37 | 0.8 |
|  | AAP | 30.02 | 99.52 | 98.78 | 0.34 | 0.73 |
|  | CTD | 23.6 | 99.57 | 98.95 | 0.27 | 0.71 |
|  | DPC | **42.18** | **99.01** | **98.41** | **0.36** | **0.84** |
|  | PCP | 11.61 | 99.89 | 99.16 | 0.23 | 0.66 |

Sen: Sensitivity; Spec: Specificity

**Table S8.** Performance of various WEKA classifiers developed using various features as input for different classes of epitopes on BalanceFix dataset.

| **Features** | **Parameter** |  | **IgG** |  |  | **IgE** |  |  |  | **IgA** |  |
| --- | --- | --- | --- | --- | --- | --- | --- | --- | --- | --- | --- |
|  |  | **SMO** | **IBk** | **RF** | **SMO** | **IBk** | **RF** |  | **SMO** | **IBk** | **RF** |
| **AAC** | **ACC** | 63.34 | 64.51 | 62.76 | 76.61 | 75.59 | 74.67 |  | 71.28 | 66.85 | 68.91 |
|  | **MCC** | 0.27 | 0.29 | 0.26 | 0.53 | 0.52 | 0.5 |  | 0.46 | 0.41 | 0.41 |
|  | **AUC** | 0.63 | 0.64 | 0.66 | 0.76 | 0.76 | 0.8 |  | 0.72 | 0.66 | 0.73 |
| **AAP** | **ACC** | 68.14 | 70.05 | 67.27 | 81.5 | 79.53 | 80.81 |  | 70.6 | 60.23 | 71.1 |
|  | **MCC** | 0.35 | 0.4 | 0.35 | 0.64 | 0.6 | 0.62 |  | 0.49 | 0.23 | 0.48 |
|  | **AUC** | 0.67 | 0.7 | 0.72 | 0.81 | 0.8 | 0.86 |  | 0.71 | 0.59 | 0.74 |
| **DPC** | **ACC** | 67.40 | 70.07 | 67.14 | 81.34 | 79.61 | 80.89 |  | 70.6 | 63.30 | 71.16 |
|  | **MCC** | 0.35 | 0.4 | 0.34 | 0.64 | 0.6 | 0.62 |  | 0.49 | 0.27 | 0.49 |
|  | **AUC** | 0.67 | 0.7 | 0.72 | 0.81 | 0.8 | 0.86 |  | 0.71 | 0.59 | 0.76 |
| **CTD** | **ACC** | 55.31 | 58.09 | 55.19 | 61.42 | 63.02 | 63.18 |  | 62.73 | 62.92 | 62.92 |
|  | **MCC** | 0.1 | 0.16 | 0.11 | 0.23 | 0.26 | 0.27 |  | 0.28 | 0.26 | 0.34 |
|  | **AUC** | 0.55 | 0.58 | 0.56 | 0.61 | 0.63 | 0.67 |  | 0.62 | 0.63 | 0.68 |
| **PHY** | **ACC** | 56.76 | 56.50 | 55.96 | 61.55 | 63.18 | 64.07 |  | 63.48 | 66.10 | 64.79 |
|  | **MCC** | 0.14 | 0.13 | 0.12 | 0.23 | 0.27 | 0.28 |  | 0.28 | 0.32 | 0.31 |
|  | **AUC** | 0.57 | 0.56 | 0.57 | 0.62 | 0.63 | 0.68 |  | 0.63 | 0.66 | 0.68 |

**Table S9.** The performance of various WEKA classifiers developed using various features as input for different classes of epitopes on BalanceVar dataset.

| **Features** | **Parameter** | **IgG** | | | **IgE** | | | **IgA** | | |
| --- | --- | --- | --- | --- | --- | --- | --- | --- | --- | --- |
|  |  | **SMO** | **IBk** | **RF** | **SMO** | **IBk** | **RF** | **SMO** | **IBk** | **RF** |
| **AAC** | **ACC** | 63.53 | 62.54 | 64.36 | 71.00 | 70.7 | 70.47 | 71.68 | 68.17 | 70.98 |
|  | **MCC** | 0.27 | 0.25 | 0.29 | 0.42 | 0.42 | 0.41 | 0.47 | 0.37 | 0.44 |
|  | **AUC** | 0.63 | 0.62 | 0.68 | 0.72 | 0.71 | 0.76 | 0.73 | 0.68 | 0.76 |
| **AAP** | **ACC** | 67.81 | 68.27 | 68.08 | 77.18 | 76.94 | 76.86 | 71.46 | 68.66 | 70.73 |
|  | **MCC** | 0.36 | 0.37 | 0.36 | 0.55 | 0.55 | 0.54 | 0.47 | 0.42 | 0.42 |
|  | **AUC** | 0.69 | 0.69 | 0.72 | 0.77 | 0.77 | 0.82 | 0.68 | 0.69 | 0.72 |
| **DPC** | **ACC** | 67.96 | 68.25 | 67.71 | 77.26 | 76.96 | 75.99 | 71.59 | 70.12 | 71.83 |
|  | **MCC** | 0.36 | 0.37 | 0.36 | 0.55 | 0.55 | 0.52 | 0.49 | 0.36 | 0.44 |
|  | **AUC** | 0.68 | 0.69 | 0.73 | 0.77 | 0.77 | 0.82 | 0.71 | 0.70 | 0.76 |
| **CTD** | **ACC** | 62.5 | 60.96 | 59.98 | 64.78 | 63.12 | 62.95 | 65.61 | 64.51 | 66.34 |
|  | **MCC** | 0.25 | 0.22 | 0.2 | 0.3 | 0.26 | 0.26 | 0.32 | 0.29 | 0.33 |
|  | **AUC** | 0.62 | 0.58 | 0.63 | 0.65 | 0.63 | 0.67 | 0.65 | 0.65 | 0.71 |
| **PHY** | **ACC** | 63.23 | 60.41 | 62.04 | 62.44 | 60.72 | 64.2 | 66.1 | 65.98 | 68.66 |
|  | **MCC** | 0.27 | 0.21 | 0.24 | 0.25 | 0.22 | 0.31 | 0.32 | 0.32 | 0.37 |
|  | **AUC** | 0.63 | 0.59 | 0.65 | 0.62 | 0.61 | 0.71 | 0.66 | 0.65 | 0.74 |
